# Why good work in philosophical bioethics often looks strange

**DOI:** 10.1007/s11017-022-09601-3

**Published:** 2022-12-06

**Authors:** Ole Martin Moen

**Affiliations:** Oslo Metropolitan University, Oslo, Norway

**Keywords:** Bioethics methodology, Philosophical bioethics, Lazy iconoclasm, Thought experiments, John Stuart Mill, Florence Nightingale

## Abstract

Papers in philosophical bioethics often discuss unrealistic scenarios and defend controversial views. Why is that, and what is this kind of work good for? My aim in the first part of this paper is to specify how philosophical bioethics relates to other types of work in bioethics, and to explain the role of the unrealistic scenarios and the controversial views. In the second part, I propose three strategies for doing research in philosophical bioethics that makes a valuable contribution to the bioethics community at large.

## Introduction

Researchers come to bioethics from a variety of disciplines, such as medicine, nursing, biology, law, health economics, health leadership, public policy, and philosophy. I come to bioethics from *philosophy*, so unsurprisingly, most of my work is concerned with the more philosophical side of bioethical inquiry, or what we might call *philosophical bioethics*.

It has become my impression, increasingly, that a significant number of researchers who come to bioethics from other disciplines find much of what goes on in philosophical bioethics strange.

There are, I think, two common features of philosophical bioethics that, understandably, can make research in this area look strange from the outside.

The first feature is that philosophers working in bioethics often discuss decision-making in contrived scenarios that bear little resemblance to practical reality in the healthcare system. For example, in one of the most cited works in twentieth century philosophical bioethics, Judith Jarvis Thomson discusses in detail a case in which a random patient, one day, wakes up to find that an artist has been connected to their bloodstream, and learns that the artist must remain connected for the next nine months for the artist to survive. Would it be permissible, Thomson asks, to unplug in this scenario [1]? This, moreover, is certainly not the only contrived example in philosophical bioethics. Reading research papers in philosophical bioethics, one will frequently come across scenarios involving technologies *far* out of reach and assessments of potential policies that have no chance at all of being implemented.

The second strange-looking feature of philosophical bioethics is that a large number of researchers in this area – indeed, many of its most central theorists – defend views that are highly counterintuitive and controversial. Among the members of the editorial board of the journal *Bioethics *– a leading journal for philosophical bioethics – we find philosophy professors who suggest that doping should be permitted in sports, who advocate for the creation of genetically engineered posthumans, who maintain that infanticide is only slightly worse than abortion, and who claim that dignity is bioethically irrelevant [2].

My aim, in what follows, is first to try to make sense of philosophical bioethics: to specify how philosophical bioethics relates to other types of work in bioethics and to explain why the unrealistic scenarios and the controversial views both play very important roles in philosophical bioethics. I then proceed to propose three strategies for doing research in philosophical bioethics that is valuable to the bioethics community at large.

## Making sense of philosophical bioethics

### Zooming out from clinical decision-making

Let me start by trying to locate philosophical bioethics within the terrain of bioethical research more broadly.

One way of mapping the terrain that is useful for the present purposes, is according to how close the decision-making that is being discussed is to individual patients in the healthcare system. Closest to individual patients is *clinical bioethics*, which is concerned with ethical decisions at the clinical (“bedside”) level. Research articles in clinical bioethics might discuss questions such as how to respect autonomy in early onset dementia patients, ethical challenges in pediatric cases where the treatment decision of parents are contrary to what is in the best interest of the child, or the ethical challenges in decision-making of nurses when the patient is a prison inmate.

One step removed from ethical decision-making in the clinic is ethical decision-making at the level of those who form guidelines and policies, which we might call *policy bioethics*. Research articles at this level might discuss, for example, ethical problems related to the influence of lobby groups, questions about fairness in allocating costly medications, or in what contexts vaccine mandates for healthcare workers might be justified.

Finally, at an even more general level, there are questions of *philosophical bioethics*. Philosophical bioethics is concerned with the overall aims of healthcare and the criteria by which policies ought to be assessed.

The aims of healthcare might seem pretty straightforward. Shouldn’t we, after all, work to promote obviously good aims such as health, happiness, respect, autonomy, and work against things such as suffering, illness, disrespect, injustice? I think all philosophical bioethicists would agree that, in a broad sense, this is indeed what we should.

Philosophical bioethicists would, however, insist that this leaves many crucial questions unanswered. What is the right tradeoff between losses in a patient’s welfare and a shortening of that patient’s life? Is there a limit to how much more negative effects we should accept if it results from an omission than from an action? Is that even the right way to pose that question? Is it always wrong to help a patient end their life? What, ultimately, constitutes a patient’s welfare? Is patient autonomy truly a good in itself, or is it a good only insofar as it promotes welfare? Is “naturalness” a relevant criterion in bioethical decision-making? Philosophical bioethicists work on questions such as these as they arise in healthcare and the biosciences.

Another question that belongs to the domain of philosophical bioethics is the following: Are we currently making serious mistakes, not just at the level of implementation, but also about the very values and considerations that guide our decision making in healthcare and the biosciences?

It seems clear that mistakes of this kind are not just possible, but that they have been made on a large scale in the relatively recent past. In the United States in 1960, it was legal in all fifty states to lobotomize psychiatric patients. Lobotomy could be initiated – as could most forms of medical treatment – at the doctors’ discretion, without the law requiring informed consent. In the same fifty states, abortion was a criminal offense, even in cases where the pregnancy was due to rape. *The American Psychiatric Association* still classified homosexuality as a disorder and recommended conversion therapy. There were no federal laws that protected the welfare of animals used in medical experiments.

Here is a chilling question: Are we, sixty years later, still guilty of grave, systematic moral wrongdoing? It might appear, intuitively, that we are not, but presumably, it rarely seemed to people in the past that they were in fact taking part in grave, systematic moral wrongdoing. Philosopher Evan G. Williams points out, in the paper “The Possibility of an Ongoing Moral Catastrophe”, that “most other societies in history … have been unknowingly guilty of serious wrongdoing,” and he suggests that this gives us a reason to suspect that we are as well. We do not, any more than previous generations, live at the end of history, and although we are hopefully on the right track on more and more issues, there still is a very “large number of distinct ways in which our practices could turn out to be horribly wrong” [3, p. 971].

It belongs to the area of philosophical bioethics to be on the outlook for ways in which we might, within the sphere of healthcare and the biosciences, be making serious mistakes of which we are currently not aware.

A formulation that I think is both simple and precise is this one: that philosophy in general is concerned with seeking to ensure that we are getting the basics right in general, and that philosophical bioethics is concerned with seeking to ensure that we are getting the basics right in bioethics.

Let us now turn to the role of unrealistic scenarios and controversial views in philosophical bioethics.

### Why the unrealistic scenarios?

To understand the reasons for entertaining unrealistic scenarios, consider, first, the well-known practice, in biomedical science and beyond, of finding out what we have most reason to believe, regarding empirical matters, by gathering data from observation, forming hypotheses, and testing hypotheses by means of controlled experiments.

Can we use physical experiments, not just for deciding what empirical premises to accept, but also for deciding what normative premises to accept? That, unfortunately, is not something that we are capable of doing. We can test, empirically, what people report to believe and to investigate how their normative judgments play out in different cases, but although such findings can give us valuable insights, finding out what people believe about normative issues is not the same as finding out what views we *should* use to guide our decision-making. Presumably, most people would, if asked in 1960, have reported that homosexuality is immoral. Today, most who read this are presumably grateful that, back then, there were people who did not take the majority view to be decisive, and that there were enough of these people to bring about a broad shift, in medicine and beyond, on the moral status of homosexuality.

One of the central methods for assessing normative premises is *thought experiments*. Why are these helpful? Consider Thomson’s thought experiment described above, in which it was considered whether it would be morally permissible to unplug in case one day, one found that an artist had been connected to one’s bloodstream and they needed to remain connected for nine months in order to survive.

Importantly, Thomson did not discuss this thought experiment because she thought that it was important that healthcare workers should be prepared in case this situation ever occurs. Rather, she formulated this thought experiment in response to the abortion debate in the 1960s. In this debate it was widely assumed – among both those who defended abortion and those who opposed it – that the crucial question in the debate about the moral legitimacy of abortion was the question of whether a fetus qualifies as a person. Thomson challenged this assumption by suggesting an example where it seems that *even if* someone who is definitely a person (the artist) would die if removed from one’s circulatory system, it would nevertheless not be clear that one would have a moral duty to keep them there over an extended period of time. Although it might be commendable to save the artist’s life by letting them stay connected for nine months, it is not clear that one would have a moral duty to do so or that it should be a criminal offense to disconnect.

As Thomson herself emphasises, this example alone is clearly not sufficient to settle the debate about the moral legitimacy of abortion. Arguably, however, the example is sufficient to show that it takes more than establishing that a fetus is a person to establish that abortion is immoral and/or should be a criminal offense.

Why are thought experiments often so contrived? The short answer is that they are contrived for the same reason that empirical experiments are contrived: In order to discover the impact of changing the one variable that one is putting to the test, one must create conditions under which it is possible to change this specific variable without also changing other variables, and this type of variable isolation is not very common in everyday situations. Consider one of the most famous experiments in the history of science: Galileo’s experiment of dropping an iron ball from the top of the mast of a ship sailing at a stable speed, where it could be observed that, despite the speed of the ship, the ball fell parallel to the mast.

Here experienced sailors might well have objected that the scenario is ridiculously unrealistic: It hardly ever happens that iron balls fall from the top of the mast, and it is a waste of time to figure out where exactly such a ball would land. What Galileo was interested in, however, was not to help sailors prepare for this unrealistic scenario: He dropped the ball in order to show that the laws of motion are the same in all inertial frames, an implication of which is that balls falling straight down on earth is not evidence that earth stands still – an issue of fundamental importance for our understanding of the laws of physics, and in turn, also for navigation [4].

Thought experiments, like physical experiments, are devised to isolate factors, which in the case of philosophical bioethics are usually normative premises. The reason why these are thought experiments, rather than physical experiments, is that we have no way to determine whether to accept or reject a normative premise by means of physical experiments.

In response to this, some might say that, as a matter of principle, we should not accept any premise unless it can be verified by empirical observation. There are, however, several well-known problems with this response. One problem is that it would commit us to give up on reasoning about normativity, as well as reasoning about lots of issues in mathematics and physics. Another problem is that it is unclear if this response can even be consistently held, for the view that “we should not accept any premise unless it can be verified by empirical observation” is itself a view (indeed, a normative view) that cannot be verified by empirical observation.

So to sum up: We need unrealistic scenarios in philosophical bioethics because we need thought experiments where we isolate individual factors in order to put our normative premises to the test.

### Why the controversial views?

Why is there a high frequency of controversial views in philosophical bioethics? To understand the mechanism at work, let us use, precisely, a *thought experiment*. Imagine that you have a month dedicated to making a valuable contribution – in the form of a research paper – to the field of philosophical bioethics. In order to do so, you have thought hard about some of the problems in philosophical bioethics. Let us say, moreover, that you have arrived at a tentative conviction about two issues. On the issue where you are most certain (let us call this Conviction #1), you feel almost fully certain. Where you are second most confident (Conviction #2) you are not fully certain, but having thought it over, you are fairly confident.

After weeks of thinking these issues through, it is now time for you to produce a paper in philosophical bioethics where you explain your reasoning. What issue should you write about? It is tempting, immediately, to write about Conviction #1, since here you are confident that your reasoning is solid. You decide, however, that your level of confidence is not your only concern; you are also concerned with using the occasion to make a positive difference to the ongoing debate and, if possible, to society. When you examine what people are proposing, you find that Conviction #1 is held by virtually everyone already. Your reasoning, moreover, is already stated in the literature. You find that Conviction #2, however, is widely rejected, and it seems that the reasons that you have found to be weighty have not been adequately considered.

Which of the convictions should you write about? It seems that you should write about Conviction #2. This is, after all, the issue on which you have reason to believe that, in your best judgment, you are likely to cast light on an issue that hasn’t hitherto been cast light on by many others. By making this choice, however, you would – alas! – thereby have made yourself a defender of a view that is widely considered controversial. This selection mechanism is crucial to understanding the high frequency of controversial views in research papers in philosophical bioethics.

We have reason to be glad that the people who were pondering over ethical issues at earlier points in history did not say just what others said but spoke up on the issues where they thought their contemporaries were mistaken. The more we are convinced by Williams’ proposal that we are likely to still be making serious moral mistakes, the stronger are our reasons to be on the outlook for such mistakes today.

Although many philosophical bioethicists defend convictions that, in most people’s view, look radical, all philosophical bioethicists that I know hold mainstream views on most bioethical issues. These are views, however, that they have much less reason to spend their research time writing about. It is also worth considering what clearly uncontroversial papers in philosophical bioethics would be like. Presumably, they would have titles like “The use of anesthesia during surgery is morally permissible,” “Doctors should not discriminate against left-handed patients,” “Toddlers should be given access to oxygen even in cases where one parent says no,” or “Care is important.”

It does not follow from this that it would be good at all if there was an equally high frequency of controversial views in clinical and policy level bioethics. Although researchers in these areas might also encounter problems of a more foundational nature, their work would, to the extent that their work focused specifically on that foundational issue, belong on the philosophical side of bioethics.

## Three suggestions for better philosophical bioethics

### Flag philosophical work as philosophical

Since researchers working in bioethics discuss decision-making at different levels – from those on the bedside to decisions about what fundamental values are worth pursuing in the health sciences – a way to avoid misinterpretation is to state explicitly on what level one is engaging. If, as someone who works in philosophical bioethics, one does not do this, it might be read by someone who works in clinical or policy level bioethics as if you are doing the same type of work that they are doing, and that you are not doing it very well.

I think philosophers approaching a bioethical issue should ask themselves if the argument that they wish to present is primarily a contribution to bioethics, or if they are really contributing to normative ethics and make use of a thought experiment from the sphere of medicine in order to make that point. If it is the latter, the paper probably belongs in a philosophy journal. One example of such a thought experiment, which does not really belong in bioethics, is Judith Jarvis Thomson’s transplant case:

Imagine yourself to be a surgeon, a truly great surgeon. Among other things you do, you transplant organs, and you are such a great surgeon that the organs you transplant always take. At the moment you have five patients who need organs. Two need one lung each, two need a kidney each, and the fifth needs a heart. If they do not get those organs today, they will all die; if you find organs for them today, you can transplant the organs and they will all live. But where to find the lungs, the kidneys, and the heart? The time is almost up when a report is brought to you that a young man who has just come into your clinic for his yearly check-up has exactly the right blood-type, and is in excellent health. Lo, you have a possible donor. All you need do is cut him up and distribute his parts among the five who need them. You ask, but he says, “Sorry. I deeply sympathize, but no.” Would it be morally permissible for you to operate anyway?” [5, p. 1396]

Insofar as it is interpreted as a work in clinical bioethics, this example is preposterous: Surely, we do not want to give surgeons the mandate to act in such a way! That, however, is not the context in which the thought experiment is raised. It is a thought experiment raised in response to Philippa Foot’s trolley case thought experiment, in which it is asked if it would be right to divert a runaway trolley to a track where it would kill one if the only alternative is to let it continue forward on a track where it would kill five [6]. Foot’s trolley case experiment, moreover, is a thought experiment that, in virtue of isolating other variables, sharply illustrates the issue of our responsibility for acts compared to our responsibility for omissions. That is a question for normative ethics. When Thomson introduces the transplant case in response to Foot’s trolley case, her point is that we need an explanation of why it appears to be permissible to divert the trolley but certainly not permissible to harvest the organs, even though the consequence of inaction (that five die) and the consequence of action (that one dies instead) are equal in both cases. That is also a question for normative ethics.

Admittedly, it might be tempting for a philosopher who makes a point in normative ethics by means of a thought experiment from medicine to try to publish their work in a bioethics journal. The best bioethics journals are nowhere near as selective as the best philosophy journals. Making work in normative ethics appear as work in bioethics, however, should be resisted by philosophers and discouraged by journal editors. A paper discussing Thomson’s transplant example does not belong in *Journal of Clinical Ethics* for the same reason that a paper discussing Foot’s trolley example does not belong in *International Journal of Rail Transportation*.

### Avoid lazy iconoclasm

Good papers in philosophical bioethics give us reason to update our thinking about broad issues, principles, values, and priorities in bioethics. To promote this type of good work, individual researchers and editors alike should work against a problem that I shall dub *lazy iconoclasm*. Lazy iconoclasm is to make a paper appear to do a lot more argumentative work than it is in fact doing.

There are different variants of lazy iconoclasm. One variant is to hide, or underplay, the extent to which one’s argument for a revisionist conclusion presupposes a controversial premise that, in fact, is doing all of the argumentative work. One example of such a work is Timothy Hsiao’s paper “Industrial Farming is Not Cruel to Animals.” Here Hsiao challenges a widely shared view in animal ethics, namely that industrial farming is cruel to animals. The argumentative work that he does is minimal, however, the reason for which is that he rests his case for this conclusion on the general premise that “animal suffering is not morally salient, and that animals lack the required features necessary for membership in the moral community” [7, p. 38]. It is not enlightening to learn that, given the premise that *nothing at all* counts as being cruel to animals, neither does industrial farming.

A related variant of lazy iconoclasm is to understate the extent to which one’s argument is dependent on a line of conditionals (“if we grant that…”, “insofar as…”, “in the cases considered here…”) that make the end conclusion much more tentative than it might first appear. Admittedly, any argument depends on restrictions and conditionals to some extent. It is crucial to the amount of argumentative work the paper is doing how much these conditionals, in sum, restrict one’s position. It is detrimental to the reputation of philosophical bioethics if a conclusion appears defended, and gains attention from doing so, even though the author, upon scrutiny, retracts to having only made the case for an uncommitting conditional [8].

One can also be guilty of lazy iconoclasm in virtue of hiding, or underplaying, the limitations on the scope of actual cases to which one’s argument applies. A way of doing that is to over-exploit this type of response to an objection: to say that although the objection might well be a weighty objection against a practice in *some* contexts, it does not rule the practice out in principle, and therefore, that it is still an open question whether the practice itself is objectionable.

Importantly, this *can* be a fine form of responding. If one is defending the legalization of euthanasia, and one’s interlocutor objects by pointing to the dangers that would result if euthanasia were implemented in a way that is obviously irresponsible, one can rightfully reply that to object to the obviously irresponsible way of implementing euthanasia is not to object to the implementation of euthanasia *per se*. The claim that there are irresponsible ways of implementing a practice is, after all, not a very interesting claim, because there are irresponsible ways of implementing virtually any practice in the biomedical sphere. The interesting question to ask, in considering whether a practice should be introduced, is not whether there are a hundred bad ways in which it could be done (there surely are), but whether there are some good ways in which it can be done.

One engages in lazy iconoclasm, however, if all one is really saying is that there is, in theory, some possible scenarios in which a practice could be justified, and therefore, that the practice cannot be ruled out *in principle*–at least insofar as one hides the fact that this is such an uncommitting claim that it can be said about virtually any practice. There are, presumably, extraordinary circumstances in which it would not be wrong to throw a hand-grenade into a passing truck. This, however, does not give one grounds for writing a paper titled “A defense of the moral permissibility of throwing hand-grenades into passing trucks.”

### Make sure that one’s philosophizing is connected to the facts on the ground

Even though philosophical bioethics is concerned with broad and general issues, research in philosophical bioethics should nevertheless be related to individual cases on the ground. To be concerned with broad and general issues is, after all, not to be concerned with things unrelated to what is at stake in individual cases on the ground, but rather, to be concerned with the things that are present in a broad range of cases, perhaps even in *all* cases. If, as a philosopher, one writes a research paper on an issue such as surrogacy, organ donation, addiction, circumcision, disability, sex work, or gender-affirming care – i.e. an issue where there is a significant number of people with first-hand experiences, and very likely, a local or national NGO or user organization that one can easily get in touch with – one should expect that there are crucial insights to be learned from talking with the people who are directly involved. Be aware, however, of the problem of research fatigue [9], be careful not to present insights and arguments generated in minority communities as if these were your own (even if you are the first to communicate these in an academic journal), ensure that you are not making claims that will appear to hate-groups as dog-whistles, and invite to co-authorship when that is due.

In doing collaborative work with researchers from outside of philosophy, it might seem natural for academic philosophers to look to fields like health policy, health law, and medicine.

I want to suggest, however, that more academic philosophers should consider combining their skill in handling broad principles with the skills of those who have the most up-close experience with the individuals on the ground in the healthcare system, the largest group of which is *nurses*.

Nursing and philosophy are very different fields. They do, however, also have notable similarities. Despite the rapid specialization in most of academia, nursing and philosophy have both remained broad and generalist disciplines; nursing and philosophy are both disciplines in which first-person experiences are taken very seriously; and nursing and philosophy are (in good cases) both characterized by a no-nonsense attitude and an eagerness to get to the core of an issue.

Nursing and philosophy, moreover, have a history of being very forceful together in the form of the collaboration between Florence Nightingale and John Stuart Mill. Since this collaboration is not widely known, I shall describe it here in brief. Nightingale, who was the founder of professional nursing, wrote that Mill’s *Principles of Logic* (1843) had been “the forming influence” on her philosophical thinking, and she described herself as one of his “adherents” [10, p. 373]. Nightingale and Mill, moreover, exchanged letters about her writing of *Notes on Nursing* (1859), and they thereafter both worked hard to have its principles implemented. This was done in the face of weighty opposition. Nurse-led hospital management (where nurses report to a head-nurse, *not* to a medical doctor), nursing schools, and medical practice based, not on physician judgment, but on evidence from medical statistics, was fought through despite loud objections from prominent members of the medical profession.

One of the central opponents of Nightingale and Mill’s calls for reform was Samuel Osborne Habershon, the president of the *Medical Society of London* and a founder of the *Christian Medical Association*. Habershon wrote, in a presidential address in the *British Medical Journal* in 1880, that «should the fashionable mania for nursing have full sway … the physician himself would be scarcely required,” and that “if the hospital is to be continued as an institution for the skillful treatment of disease, it must be under the direction of the doctors, instead of the matron, in all things that concern the patients.” This, he stressed, included both training and authority: “The training of nurses must necessarily come from the doctors, just as right nursing must be under their control. The doctor must, in his practical knowledge of disease and its requirements, be the director in the details of nursing.” Doctors’ judgment and authority, he also held, is needed to ensure that “nurses are actuated by truly Christian principles” [11, pp. 120–21].

Nightingale was relentlessly dissatisfied with the premise that a doctor’s judgment in a case should be regarded as authoritative unless there is evidence that the judgment is right. In her view, “the greatest thinkers, the most earnest, daring seekers after truth have never used words like ‘private judgment’ at all” [12, p. 93]. She opposed the authority of the doctor’s judgment, moreover, not only on empirical matters; she also opposed the authority of the doctor’s conscience as a guide to medical ethics. “Conscience,” Nightingale wrote,

has told different nations and different ages different things. Without all the faculties which go to make up, to gather what we call experience, conscience is nothing. It tells us now to consider crimes what it told other periods to consider duties. Conscience told the old Romans to kill themselves. Conscience let the patriarchs have many wives and many concubines. Conscience told Calvin to burn Servetus. Conscience told Luther to marry a pretty nun. [12, p. 75]

Although Nightingale was not a utilitarian, she and Mill both fought for medical practice to be guided by the aim of minimizing harms from disease. Both also grew disillusioned by the arrogance and inertia of the medical establishment of their time. Medical doctors, Nightingale wrote to Mill, “do not fail in getting their own livelihood but they fail in doing good and improving therapeutics” [13, p. 376]. Mill, in his reply two days later, wrote that “my opinion of the medical profession is not, I dare say, higher than yours” [14, p. 378].

Medical practice has, of course, improved dramatically since the mid-to-late 19th century. It remains the case, however, that medical doctors, individually and collectively, wield significant power. Medical doctors earn high salaries, restrict everyone’s access to pharmaceuticals, often work to uphold severe restrictions on embryonic stem cell research, restrict competent adults from using biomedical technologies for the purposes of enhancement, and – in many countries – still work to block access to assisted dying. My point here is not to argue that any particular of these uses of power is wrongful. My point is just that medical doctors are a powerful force in society, not just on issues pertaining to medical facts, but also on questions of value, and that as a powerful force, it ought to be checked by counterpowers.

I want to suggest that today, as in the 19th century, collaboration between philosophers and nurses can be a highly impactful counterpower. Even in the absence of luminaries like Nightingale and Mill, a team of nurses and philosophers can form a powerful team in discussions of bioethical issues, because such a team combines those who work closest to individual patients with those who work with the broadest abstractions.

## Conclusion

I have had two aims in this paper. The first aim has been to help make sense of philosophical bioethics to those who come to bioethics from other disciplines, and to explain what the high prevalence of unrealistic scenarios and controversial views are good for. The unrealistic scenarios are thought experiments, which is one of the central ways of putting normative principles to the test. The controversial views are needed because, among all the issues about which a philosophical bioethicist might share her reasoning with others in their field, that which she has most reason to share (if she wishes to make a valuable contribution) is the reasoning indicating that we might currently be making a major mistake.

I have then pointed to three suggestions for more valuable work in philosophical bioethics: to flag philosophical bioethics as philosophical bioethics, so as to make sure that it is not read as, for example, clinical bioethics; to avoid the pitfall that I have dubbed lazy iconoclasm; and to actively take steps to make sure one’s work in philosophical bioethics is connected to the “facts on the ground.” The latter, I suggest, can be done through seeking input and/or collaboration with people personally affected by the issue one is working on. It can also be done through seeking collaboration with professionals in the field of nursing, since when philosophers and nurses team up – as exemplified by the collaboration of John Stuart Mill and Florence Nightingale – they have the potential to bring about much-needed change.
